# L-Menthol Olfactory Stimulation Reduced Dyspnea Sensation during the 6 min Walk Test in Patients with Chronic Breathlessness Syndrome: A Pilot Study

**DOI:** 10.3390/jcm12175587

**Published:** 2023-08-27

**Authors:** Naofumi Sato, Ryoji Ogura, Yuji Iwanami, Ikuko Okuni, Satoru Ebihara

**Affiliations:** 1 Department of Rehabilitation Medicine, Toho University Omori Medical Center, 6-11-1 Omori-nishi, Ota-ku, Tokyo 143-8541, Japan; 2 Department of Internal Medicine and Rehabilitation Science, Tohoku University Graduate School of Medicine, 1-1 Seiryo-machi, Aoba-ku, Sendai, Miyagi 980-5874, Japan

**Keywords:** dyspnea, modified Borg score, 6 min walk distance, surgical mask

## Abstract

There are very limited methods of relieving dyspnea that are independent of the causative disease. L-menthol olfactory stimulation is reported to be effective for dyspnea during exercise and inspiratory resistance. Therefore, we examined the effects of L-menthol olfactory stimulation on exertional dyspnea during the 6 min walking distance test (6MWT) in patients with chronic breathlessness syndrome. The subjects who consented to the study were divided into two groups. In Group A, the first 6MWT was performed as usual (placebo) while wearing a surgical mask, and the second 6MWT was performed under the L-menthol condition. In Group B, the first 6MWT was performed under the L-menthol condition, and the second 6MWT was performed as a placebo. A total of 16 subjects (70.8 ± 9.5 years) were included in the analysis. As for the effect of reducing dyspnea, a significant difference was observed in Group A patients who underwent the L-menthol condition in the second 6MWT (*p* = 0.034). In the comparison of the 6 min walking test under the L-menthol condition and the placebo, the modified Borg scale gain was significantly different between the L-menthol condition and the placebo (*p* = 0.007). Our results suggested that the L-menthol olfactory stimulation reduced dyspnea on exertion in patients with chronic breathlessness syndrome.

## 1. Introduction

Dyspnea is a major symptom in patients with advanced cancer or chronic obstructive pulmonary disease (COPD). There are many questions concerning optimal management and, specifically, whether various therapies are effective in each setting. Pathophysiologic treatments, such as drug therapy, are rarely able to completely treat dyspnea. When this condition persists for a long period of time, it is referred to as chronic breathlessness syndrome according to the expert consensus of the European Respiratory Society [1]. Dyspnea in chronic breathlessness syndrome is observed to worsen as the underlying disease progresses [2,3]. The appearance of dyspnea in everyday life leads to anxiety due to exertion-induced dyspnea, especially in respiratory diseases, which leads to the limitation of physical activity and depression [4,5], resulting in the deterioration of the quality of life (QOL) [6]. Furthermore, a community health survey by Currow et al. [7] reported that having chronic dyspnea was related not only to disease and age but also to the socioeconomical status of the patient, which has been suggested to be a factor. There are very limited methods of relieving dyspnea that are independent of the causative disease and/or factors.

Recently, non-pharmacological treatments such as blowing cold air onto the face [8,9] and transient receptor potential melastatin 8 (TRPM8) receptor stimulants are attracting attention as a treatment for dyspnea. TRPM8 functions as a somatosensory cold receptor that is activated by non-noxious cold stimuli below 25 °C. Furthermore, it has been reported that TRPM8 is activated not only by non-noxious cold stimuli but also by compounds that induce a cold sensation, such as L-menthol [10,11]. Kanezaki et al. reported that L-menthol olfactory stimulation is effective for dyspnea during exercise and inspiratory resistance [12]. However, it is unclear whether TRPM8 stimulants reduce exertional dyspnea in patients with chronic respiratory syndrome.

In this age, when SARS-CoV-2 is prevalent and people wear masks on a daily basis, we hypothesized that applying a TRPM8 stimulant to a mask as an olfactory stimulus would be a simple non-pharmacological treatment for dyspnea that can be used on a daily basis. Therefore, for chronic dyspnea syndrome, we performed a 6 min walking test under olfactory stimulation of a TRPM8 receptor stimulant while wearing a surgical mask in patients with chronic respiratory disease and postoperative heart disease. Thereafter, we examined the effects on exertional dyspnea and walking distance.

## 2. Materials and Methods

### 2.1. Subjects

The subjects were patients with chronic respiratory diseases and post-cardiovascular surgery patients who were referred to the rehabilitation department between August 2021 and March 2022 and consented to this study. We excluded patients who showed an adverse reaction to TRPM8 olfactory stimulation, patients with dementia, patients with orthopedic diseases that cause gait disturbance and central nervous system disease, those in a progressively worsening and unstable condition, those with obvious pulmonary hypertension, pneumomediastinum, and pneumothorax, those with other diseases (terminal stage of malignant tumor, etc.) predicted to have a poor prognosis, those deemed to be inappropriate for the study by the attending physician, and those who did not consent to this study. As ethical considerations, we explained that the patient would not suffer any disadvantages as a result of their refusal to participate in this study, that they would have a guaranteed right to refuse, and that personally identifying information would be anonymized at the time of data disclosure. Thereafter, the subjects signed the informed consent. This study was approved by the Ethics Review Board of Toho University Omori Hospital (approval number M21101).

### 2.2. Study Design

**Figure 1:**
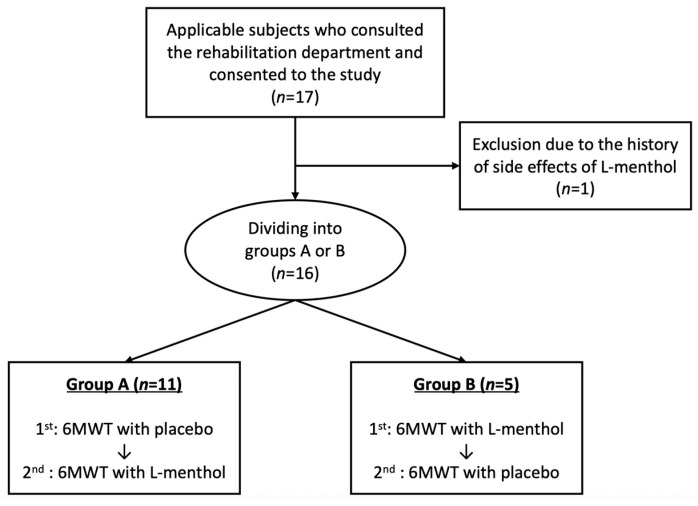
Flow of patient recruitment and protocol selection.

As a test characteristic of 6MWT, reports have claimed that it improves the 6 min walking distance (6MWD) after the second test in patients with respiratory disease [13]. Therefore, in order to prevent the influence of the learning effects, this study was designed as a crossover comparative study and was positioned as a pilot study. Figure 1 shows the research protocol. The subjects who consented to the study were divided into two groups, Group A and Group B. In Group A, the first 6MWT was performed as usual (placebo) while wearing a surgical mask, and the second 6MWT was performed under the L-menthol condition. In Group B, the first 6MWT was performed under the L-menthol condition, and the second 6MWT was performed as a placebo. The subjects were divided into two groups after confirming their preference for Group A or Group B. The two 6MWTs were evaluated on different days within one week, regardless of whether they were inpatients or outpatients. Patients who were evaluated during hospitalization were evaluated approximately one week before discharge from the hospital when their condition was stable. In our previous study in COPD, with a 20 min interval, the effects on experimental dyspnea were sufficiently washed out for menthol olfactory stimulation [14]. Moreover, the effect of menthol-flavored gum chewing was reported to be washed out with a 30 min interval on 6MWD in COPD patients [15]. We performed this study on the assumption that the effects of menthol olfactory stimulation may not carry over the course of the day.

### 2.3. L-Menthol Olfactory Stimulation

In the L-menthol olfactory stimulation, we used the commercially available Media Aroma Seal^®^ (Link Delight Ltd., Tokyo, Japan). The Media Aroma Seal^®^ is a circular patch with a diameter of 15 mm that was developed for users to smell the aroma while wearing a mask. The ingredients included eucalyptus (64.7%), peppermint (5.6%), lime (26.0%), spearmint (2.4%), and basil (1.3%). TRPM8 stimulant receptors are nociceptive cold receptors, and several stimulants are present. Among those used in Medialoma, the compounds containing TRPM8 stimulants were spearmint, eucalyptus, and peppermint. Of these, L-menthol in spearmint and peppermint has been reported to be the most potent stimulator of TRPM8 receptors [11], so L-menthol is the focus of this study. In this study, a Media Aroma Seal^®^ was attached to the inside of the surgical mask at a location that does not directly touch the lips. Before the 6MWT, we asked the subjects whether they could smell the patch and adjusted the position of the patch accordingly.Skin sensitization (allergy) and skin sensitization (with dermatitis) have been reported as side effects of TRPM8 olfactory stimulants (especially L-menthol) in very rare cases. To minimize the risk of side effects, we asked patients whether they had experienced any side effects when using products containing L-menthol, such as cooling cosmetics, food fragrances, medical poultices, and ointments.

### 2.4. Outcomes

#### 2.4.1. Primary Outcome (6MWT)

Briefly, 6MWT was performed according to the guidelines set forth by the American Thoracic Society [16]. The patients were fitted with a portable pulse oximeter (PULSOX-300, Konica Minolta Ltd., Tokyo, Japan) and instructed to walk as much as possible in 6 min along a 60 m corridor while continuously monitoring their oxygen saturation and heart rate. The 6MWT was discontinued if (a) the SpO_2_ remained below 90%, (b) if the HR was >130, <50 bpm, or increased to ≥30 bpm prior to the task, (c) upon the occurrence of a new arrhythmia, (d) upon the occurrence of subjective symptoms (dizziness, nausea, chest pain, headache, intense fatigue, cold sensation, cold sweat, and marked dyspnea), and (e) when safe monitoring could not be performed.The outcomes during implementation were the total 6 min walking distance (6MWD), oxygen saturation during 6MWT, and dyspnea at the start and end of the 6MWT using the modified Borg scale, all of which were evaluated on a scale of 0 to 10. The analysis method for each outcome measured by 6MWT is described below. In addition to the 6MWD, we calculated the ratio of the predicted value to the measured value as an index of the total walking distance (%6MWD). We used the prediction formula created by Enright [17]: {male: (7.57 × height cm) − (5.02 × age) − (1.76 × weight kg) − 309 m, female: (2.11 × height cm) − (5.78 × age) − (2.29 × body weight kg) + 667 m}. The lowest oxygen saturation (lowest SpO_2_) measured during the 6MWT was used as the oxygen saturation. The modified Borg scale gain was calculated by subtracting the modified Borg scale at rest from the modified Borg scale at the end of the 6MWT. As an index of the change in each evaluation item after 6MWT was performed twice, the amount of change (Δ) was calculated by subtracting the value from the first time to the second time.

#### 2.4.2. Secondary Outcomes

Age, sex, and severity were investigated from the medical records. The index of subjective dyspnea was graded from 0 to 4 on the Modified Medical Research Council Dyspnea scale (mMRC scale). The vital capacity (VC), expiratory capacity in 1 s, DLco, pH, PaO_2_, and PaCO_2_ were measured as respiratory functions. The data on the respiratory function indicators were mainly lacking in post-cardiovascular surgery patients, and pH, PaO_2_, and PaCO_2_ were also absent in patients with chronic obstructive pulmonary disease. The left ventricular ejection fraction was evaluated via echocardiography as an index of cardiac function. The physical function was measured not only via 6MWT but also with a hand-held dynamometer as an indicator of upper limb muscle strength, grip strength, and lower limb muscle strength. Lower limb strength was estimated by measuring isometric knee extension strength with a hand-held dynamometer (a Mobie: Sakai Medical Corp., Tokyo, Japan). Patients sat on a training bench and adjusted the position of their gluteal region so that a bench leg was behind the lower extremity on the measurement side. We performed measurements three times for each leg at intervals of 30 s. The largest value was used to calculate the ratio of knee extension strength to body weight. Peak grip strength was assessed for each hand with the shoulder and wrist in neutral positions. We performed measurements three times for each hand, and the largest value was used as grip strength.

### 2.5. Statistical Processing

The Shapiro–Wilk test was used to confirm the normality of each outcome measured by the 6MWT. As a result, there were items with *p* > 0.05 for more than one outcome. Therefore, the Wilcoxon signed rank sum test was used to compare the first and second 6MWTs before and after and to compare each evaluation item according to the implementation conditions. The Mann–Whitney U test was used to compare the amount of change between groups. Spearman’s rank correlation coefficient was used to examine the relationship between walking speed and breathlessness. SPSS version 28.0 (SPSS Inc., Chicago, IL, USA) was used as statistical software, and the significance level was set at 5%.

## 3. Results

Table 1 shows the basic attributes of this study. A total of 16 subjects (9 men, aged 70.8 ± 9.5 years) were included in the analysis. Of the 13 patients with chronic respiratory disease, 10 patients had interstitial pneumonia, and 3 patients had chronic obstructive pulmonary disease. Three patients had undergone cardiovascular surgery. As for the preoperative diagnosis, one patient had unstable angina pectoris, one patient had aortic dissection, and one patient had tetralogy of Fallot. There were no COVID-19-affected patients in the study population.

None of the 16 subjects subjected to analysis experienced any adverse events during, before, or after the 6 min walk test in this study.

Table 2 shows the results of each outcome during the first and second 6MWT. As for the effect of reducing dyspnea due to the olfactory stimulation of the TRPM8 stimulant, a significant difference was observed in Group A patients who underwent the L-menthol condition in the second 6MWT (*p* = 0.034). No significant difference was observed in Group B patients who underwent the first L-menthol condition (*p* = 0.080).

Table 3 shows the results of the amount of change (Δ) in each evaluation item of Group A and Group B. A significant difference was observed only in the ΔModified Borg scale gain between the two groups (*p* = 0.013).

Table 4 shows the evaluation results of the 6 min walking test under the L-menthol condition and placebo. Only the modified Borg scale gain was significantly different between the L-menthol condition and the placebo (*p* = 0.007). There were no significant differences in the 6MWD, %6MWD, lowest SpO_2_, or modified Borg scale at rest.

**Figure 2:**
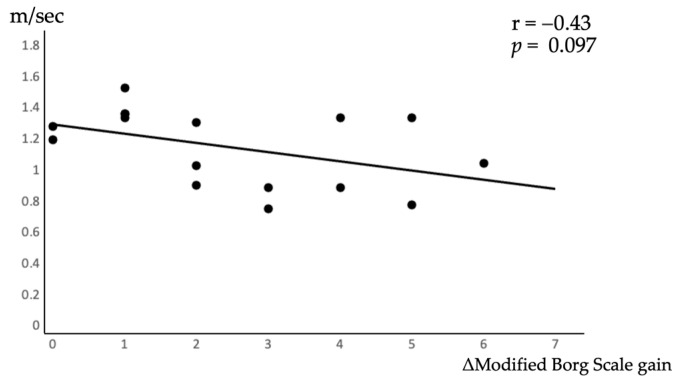
Correlation between walking speed and ΔModified Borg scale gain.

Figure 2 shows the correlation between walking speed and the ΔModified Borg scale gain. Walking speed was calculated from 6MWD without L-menthol. As a result, there was no significant correlation between walking speed and shortness of breath (r = −0.43, *p* = 0.097).

## 4. Discussion

In this study, we investigated the effects of the presence or absence of the TRPM8 olfactory stimulant on surgical masks on the 6 min walking test. As a result, we found that the addition of a TRPM8 olfactory stimulant significantly reduced the subjective dyspnea from rest to after the 6MWT (Table 4).

From Table 2, in this 6MWD, a learning effect was observed when the second 6MWT was performed. However, the learning effect was not significantly different in Group B patients who underwent the L-menthol condition in the first session. Additionally, from Table 2 and Table 3, the modified Borg scale gain in Group A, in which the second 6MWT was under the L-menthol condition, decreased at the second time compared to the first time (ΔModified Borg scale gain = −0.4 ± 0.5). Group B showed contrasting results (ΔModified Borg scale gain = 0.6 ± 0.5). Subjects also showed no relationship between walking speed and breathlessness (Figure 1), and walking speed did not contribute to the degree of breathlessness. These findings suggest that the olfactory stimulation of the TRPM8 receptor stimulant suppresses dyspnea, regardless of whether there is a learning effect in continuous 6MWT. Furthermore, the TRPM8 stimulant suppressed dyspnea, which contributed to differences in the learning effect of 6MWD between the groups.

The mechanism by which the olfactory stimulation of TRPM8 receptor stimulants relieves dyspnea during exercise has not been completely elucidated. TRPM8 is said to be expressed in the trigeminal nerve and vagus nerve among the peripheral sensory nerves [18], is activated by smell and deposition on the nasal and orbital mucosa, and can be perceived as a cold stimulus. Sensing models of dyspnea not only include chemoreceptors and mechanoreceptors [19], but various factors are involved, such as the projection of sensations within the central nervous system and the mismatch between the motor output from the motor cortex and the actual amount of movement (ventilation) [20]. As a mechanism for reducing dyspnea in this study, it is conceivable that it contributed to the correction of the mismatch between the exercise output and the actual amount of exercise. By transmitting hypoxia due to the exercise to the respiratory center, the respiratory center issues a command to increase the respiratory muscle activity as the ventilation increases. However, dyspnea is thought to occur when there is a discrepancy or mismatch between the output from the respiratory center and the afferent input from the peripheral nerve receptors. It has been previously reported that upper airway cooling or the stimulation of upper airway TRPM8 receptors inhibits respiratory muscle activity in both humans and animals [21,22,23]. Furthermore, TRPM8 is thought to play a role as a biological airway flow sensor [10,11,24]. Therefore, it is possible that the activation of TRPM8 artificially reduced the respiratory effort, modified the ventilatory response, corrected the mismatch with respiratory motor output, and contributed to the reduction in subjective dyspnea.

Furthermore, correcting the mismatch with the respiratory motor output via TRPM8 activation leads to the alleviation of unpleasant stimuli, such as dyspnea; therefore, it is thought that the main effect is psychological. Galbraith et al. used a hand fan to blow air onto 50 of her COPD and heart disease patients requiring palliative care and reported that the facial air flow reduced dyspnea [9]. In addition, Wong et al. reported that there was no change in the respiratory rate or SpO_2_ after blowing air with a hand fan, and only dyspnea was significantly reduced in terminal cancer patients [25]. In the present study, the only significant change during 6MWT was the subjective symptom of dyspnea, and there was no effect on physical functions such as the lowest SpO_2_ and total walking distance.

The minimum clinical change (MCID) for dyspnea is 0.2–2.0 for chronic heart failure [26] and 2.0 for chronic obstructive pulmonary disease [27]. In this study, the mean difference in the Δ-corrected Borg between the placebo and L-menthol groups was 0.5, which is below the MCID in respiratory disease. Therefore, we cannot expect a significant alleviation of respiratory distress in daily life. However, since such a simple method as attaching a patch to a mask leads to a considerable reduction in dyspnea, we believe that it is effective in suppressing the anxiety caused by dyspnea and improving the restrictions on physical activity.

The primary limitation of this study is that the grouping was not performed randomly. At first, we tried to randomly assign subjects, but we could not gather enough subjects. However, it was not possible to group the populations of each group with an equal number of individuals. Therefore, we decided to let subjects choose the order of menthol intervention in this study. The second limitation is that the target disease was chronic breathlessness syndrome; the mixture of respiratory and cardiovascular diseases and the mechanisms that induce dyspnea in respiratory and cardiovascular diseases might be different [28,29,30]. When we performed a subanalysis in 13 patients with respiratory diseases, we failed to obtain any significant results in comparison of the first and second 6MWTs (the same analysis with Table 2), comparison of the change in the 6MWT indices between Groups A and B (the same analysis with Table 3), and comparison between the 6MWTs with placebo and L-menthol (the same analysis with Table 4). We warrant a further larger sample study to clarify the effect of menthol on this specific disease.

Furthermore, Tsutsumi et al. reported that the ingestion of an L-menthol solution relieved dyspnea during exercise and extended the running time in competitive runners [31]. Therefore, there is room to reconsider the addition method for TRPM8 receptor-stimulating substances. Other limitations of the study are that the period between each visit for 6MWD varies and the subjects are a mixture of inpatients and outpatients. These variations could be confounding factors when interpreting the results. Further studies are required to elucidate these issues.

In analyzing the mechanism, this study has the limitation that the stimulation cannot be precisely controlled in a receptor-specific manner. Although the material in this study seems to mainly stimulate TRPM8, other receptors/channels are also stimulated at the same time. Therefore, the results may not be due to the stimulation of TRPM8 alone. In addition, as another limitation, it was not clearly elucidated that the time required for the effect of olfactory stimulation on the 6 min walk test to be completely washed out. In this study, we reluctantly proceeded based on the assumption that the effects of menthol olfactory stimulation may not carry over the course of the day. Additional experiments were warranted to evaluate the interval to wash out the effect of menthol and/or other scents’ olfactory stimulations.

Our results suggested that L-menthol olfactory stimulation reduced dyspnea on exertion in patients with chronic breathlessness syndrome. Further studies are warranted to evaluate the effects of L-menthol olfactory stimulation on exercise performance and related benefits in patients who complain of dyspnea.

## Figures and Tables

**Table 1:** Characteristics of the subjects.

	*n*	The Values Are Mean ± SD
Age (years)	*n* = 16	70.8 ± 9.5
Sex (Male/Female)	*n* = 16	9/7
BMI ^1^ (kg/m^2^)	*n* = 16	22.6 ± 3.2
Disease	*n* = 16	
(Respiratory/Cardiovascular)	(13/3)	
Classification		
IP ^2^	*n* = 10 (IPF/NSIP/CVD-ILD = 2/5/3)	
COPD ^3^	*n* = 3	
Post-cardiovascular surgery	*n* = 3	
Severity		
GAP Index (I/II/III)	*n* = 10	5/3/2
GOLD (I/II/III/IV)	*n* = 3	2/1/0/0
NYHA (I/II/III/IV)	*n* = 3	2/0/1/0
mMRC (0/1/2/3/4)	*n* = 12	4/4/2/1/1
Pulmonary function		
FVC (L)	*n* = 13	2.6 ± 0.9
%FVC (%predicted)	*n* = 13	89.5 ± 28.0
FEV_1_ (L)	*n* = 13	2.0 ± 0.7
%FEV_1_ (% predicted)	*n* = 13	89.9 ± 32.8
FEV_1_/FVC (%)	*n* = 13	78.3 ± 11.9
DLco (%predicted)	*n* = 13	75.9 ± 34.5
pH	*n* = 9	7.5 ± 0.2
PaO_2_ (mmHg)	*n* = 9	93.4 ± 25.1
PaCO_2_ (mmHg)	*n* = 9	42.0 ± 6.3
Cardiac function		
LVEF (%)	*n* = 16	70.8 ± 6.6
Muscle strength		
Quadriceps force (Nm/kg)	*n* = 12	1.4 ± 0.5
Handgrip strength (kg)	*n* = 12	26.8 ± 9.9

^1^ BMI, body mass index; ^2^ IP, interstitial pneumonia; ^3^ COPD, chronic obstructive pulmonary disease; GAP Index, gender-age-physiology; GOLD, Global Initiative for Chronic Obstructive Lung Disease; NYHA, New York Heart Association; mMRC, modified British Medical Research Council questionnaire; FVC, forced vital capacity; FEV_1_, forced expiratory volume in 1.0 s; DLco, diffusing capacity of the lung for carbon monoxide; pH, potential hydrogen; PaO_2_, partial pressure of arterial oxygen; PaCO_2_, partial pressure of arterial carbon dioxide; LVEF, left ventricle ejection fraction.

**Table 2:** Results of the first and second 6MWTs.

	Total (A and B) (*n* = 16)			Group A (*n* = 11)			Group B (*n* = 5)		
	1st 6MWT	2nd 6MWT	*p*	1st 6MWT	2nd 6MWT	*p*	1st 6MWT	2nd 6MWT	*p*
6MWD (m)	404.1 ± 97.0	430.9 ± 89.0	0.010 *	433.2 ± 88.2	460.9 ± 80.3	0.012 *	340 ± 91.7	365.0 ± 76.8	0.465
%6MWD (%predicted)	81.6 ± 24.3	87.0 ± 22.5	0.008 *	86.6 ± 26.4	91.7 ± 24.0	0.012 *	70.8 ± 20.0	76.9 ± 16.4	0.465
Lowest-SpO_2_ (%)	90.6 ± 5.0	90.4 ± 5.4	0.375	90.4 ± 4.2	90.0 ± 4.6	0.339	91.0 ± 7.0	91.2 ± 7.6	0.705
Modified Borg scale (at rest)	0.25 ± 0.7	0.25 ± 0.7	1.000	0.4 ± 0.8	0.4 ± 0.8	1.000	0.0± 0.0	0.0 ± 0.0	1.000
Modified Borg scale gain	2.3 ± 1.7	2.2 ± 1.8	0.651	1.8 ± 1.6	1.4 ± 1.4	0.034 *	3.4 ± 1.6	4.0 ± 0.6	0.083

Group A: placebo → L-menthol, Group B: L-menthol → placebo. Data analyzed via the Wilcoxon signed-rank test. The values are mean ± SD. *: *p* < 0.05. 6MWD, 6 min walk distance; %6MWD, 6 min walk distance % predicted; SpO_2_, percutaneous oxygen saturation.

**Table 3:** Comparison of changes in the 6MWT indices between Groups A and B.

	Group A (*n* = 11)	Group B (*n* = 5)	*p*
Δ6MWD (m)	27.7 ± 28.2	25.0 ± 49.5	0.441
Δ%6MWD (% predicted)	5.0 ± 4.9	6.1 ± 11.4	0.510
ΔLowest-SpO_2_ (%)	−0.3 ± 1.4	0.2 ± 2.2	0.913
ΔModified Borg Scale gain	−0.4 ± 0.5	0.6 ± 0.5	0.013 *

Group A: placebo → L-menthol, Group B: L-menthol → placebo. Data analyzed via Mann–Whitney U-test. The values are mean ± SD. *: *p* < 0.05. 6MWD, 6 min walk distance; 6MWD, 6 min walk distance; %6MWD, 6 min walk distance % predicted; SpO_2_, percutaneous oxygen saturation.

**Table 4:** Comparisons between the 6MWT with placebo and L-menthol (n = 16).

	Placebo	L-Menthol	*p*
6MWD (m)	411.4 ± 88.0	423.1 ± 99.5	0.136
%6MWD (% predicted)	84.1 ± 25.1	88.4 ± 23.7	0.158
Lowest-SpO_2_ (%)	90.6 ± 5.2	90.3 ± 5.2	0.404
Modified Borg Scale (at rest)	0.25 ± 0.7	0.25 ± 0.7	1.000
Modified Borg Scale gain	2.5 ± 1.9	2.0 ± 1.7	0.007 *

Data analyzed by the Wilcoxon signed-rank test. The values are mean ± SD. *: *p* < 0.05. 6MWD, 6 min walk distance; 6MWD, 6 min walk distance; %6MWD, 6 min walk distance % predicted; SpO_2_, percutaneous oxygen saturation.

## Data Availability

The data presented in this study are available on request from the corresponding author.

## References

[1] Johnson M.J., Yorke J., Hansen-Flaschen J., Lansing R., Ekström M., Similowski T., Currow D.C. (2017). Towards an expert consensus to delineate a clinical syndrome of chronic breathlessness. Eur. Respir. J..

[2] Currow D.C., Smith J., Davidson P.M., Newton P.J., Agar M.R., Abernethy A.P. (2010). Do the trajectories of dyspnea differ in prevalence and intensity by diagnosis at the end of life? A consecutive cohort study. J. Pain. Symptom Manag..

[3] Ahmadi Z., Lundström S., Janson C., Strang P., Emtner M., Currow D.C., Ekström M. (2015). End-of-life care in oxygen-dependent COPD and cancer: A national population-based study. Eur. Respir. J..

[4] De Peuter S., Janssens T., Van Diest I., Stans L., Troosters T., Decramer M., Van den Bergh O., Vlaeyen J.W. (2011). Dyspnea-related anxiety: The Dutch version of the Breathlessness Beliefs Questionnaire. Chron. Respir. Dis..

[5] Janssens T., De Peuter S., Stans L., Verleden G., Troosters T., Decramer M., Van den Bergh O. (2011). Dyspnea perception in COPD: Association between anxiety, dyspnea-related fear, and dyspnea in a pulmonary rehabilitation program. Chest.

[6] Gruenberger J.B., Vietri J., Keininger D.L., Mahler D.A. (2017). Greater dyspnea is associated with lower health-related quality of life among European patients with COPD. Int. J. Chron. Obstruct Pulmon Dis..

[7] Currow D.C., Plummer J.L., Crockett A., Abernethy A.P. (2009). A community population survey of prevalence and severity of dyspnea in adults. J. Pain. Symptom Manag..

[8] Schwartzstein R.M., Lahive K., Pope A., Weinberger S.E., Weiss J.W. (1987). Cold facial stimulation reduces breathlessness induced in normal subjects. Am. Rev. Respir. Dis..

[9] Galbraith S., Fagan P., Perkins P., Lynch A., Booth S. (2010). Does the use of a handheld fan improve chronic dyspnea? A randomized, controlled, crossover trial. J. Pain. Symptom Manag..

[10] Peier A.M., Moqrich A., Hergarden A.C., Reeve A.J., Andersson D.A., Story G.M., Earley T.J., Dragoni I., McIntyre P., Bevan S. (2002). A TRP channel that senses cold stimuli and menthol. Cell.

[11] McKemy D.D., Neuhausser W.M., Julius D. (2002). Identification of a cold receptor reveals a general role for TRP channels in thermosensation. Nature.

[12] Kanezaki M., Ebihara S. (2017). Effect of the cooling sensation induced by olfactory stimulation by L-menthol on dyspnoea: A pilot study. Eur. Respir. J..

[13] Singh S.J., Puhan M.A., Andrianopoulos V., Hernandes N.A., Mitchell K.E., Hill C.J., Lee A.L., Camillo C.A., Troosters T., Spruit M.A. (2014). An official systematic review of the European Respiratory Society/American Thoracic Society: Measurement properties of field walking tests in chronic respiratory disease. Eur. Respir. J..

[14] Kanezaki M., Terada K., Ebihara S. (2020). Effect of Olfactory Stimulation by L-Menthol on Laboratory-Induced Dyspnea in COPD. Chest.

[15] Prieur G., Beaumont M., Delorme M., Combret Y., Medrinal C., Hilfiker R., Bonnevie T., Gravier F.E., Smondack P., Lamia B. (2021). Short-term effects of menthol on walking dyspnoea in patients with COPD: A randomised, single blinded, cross-over study. ERJ Open Res..

[16] ATS Committee on Proficiency Standards for Clinical Pulmonary Function Laboratories (2002). ATS statement: Guidelines for the six-minute walk test. Am. J. Respir. Crit. Care Med..

[17] Enright P.L., Sherrill D.L. (1998). Reference equations for the six-minute walk in healthy adults. Am. J. Respir. Crit. Care Med..

[18] Plevkova J., Kollarik M., Poliacek I., Brozmanova M., Surdenikova L., Tatar M., Mori N., Canning B.J. (2013). The role of trigeminal nasal TRPM8-expressing afferent neurons in the antitussive effects of menthol. J. Appl. Physiol..

[19] Campbell E.J., Godfrey S., Clark T.J., Freedman S., Norman J. (1969). The effect of muscular paralysis induced by tubocurarine on the duration and sensation of breath-holding during hypercapnia. Clin. Sci..

[20] Manning H.L., Schwartzstein R.M. (1995). Pathophysiology of dyspnea. N. Engl. J. Med..

[21] Burgess K.R., Whitelaw W.A. (1984). Reducing ventilatory response to carbon dioxide by breathing cold air. Am. Rev. Respir. Dis..

[22] Burgess K.R., Whitelaw W.A. (1988). Effects of nasal cold receptors on pattern of breathing. J. Appl. Physiol..

[23] Orani G.P., Anderson J.W., Sant’Ambrogio G., Sant’Ambrogio F.B. (1991). Upper airway cooling and l-menthol reduce ventilation in the guinea pig. J. Appl. Physiol..

[24] Fisher J.T. (2011). TRPM8 and dyspnea: From the frigid and fascinating past to the cool future?. Curr. Opin. Pharmacol..

[25] Wong S.L., Leong S.M., Chan C.M., Kan S.P., Cheng H.W. (2017). The Effect of Using an Electric Fan on Dyspnea in Chinese Patients With Terminal Cancer. Am. J. Hosp. Palliat. Care.

[26] Oxberry S.G., Bland J.M., Clark A.L., Cleland J.G., Johnson M.J. (2012). Minimally clinically important difference in chronic breathlessness: Every little helps. Am. Heart J..

[27] Ries A.L. (2005). Minimally clinically important difference for the UCSD Shortness of Breath Questionnaire, Borg Scale, and Visual Analog Scale. COPD.

[28] Sullivan M.J., Higginbotham M.B., Cobb F.R. (1988). Increased exercise ventilation in patients with chronic heart failure: Intact ventilatory control despite hemodynamic and pulmonary abnormalities. Circulation.

[29] Kurosawa H., Kohzuki M. (2004). Images in clinical medicine. Dynamic airway narrowing. N. Engl. J. Med..

[30] Hansen J.E., Wasserman K. (1996). Pathophysiology of activity limitation in patients with interstitial lung disease. Chest.

[31] Tsutsumi Y., Momma H., Ebihara S., Nagatomi R. (2022). L-menthol administration facilitates breathing comfort during exhaustive endurance running and improves running capacity in well-trained runners: A randomized crossover study. Eur. J. Sport. Sci..

